# Effects of vitamin D binding protein phenotypes and vitamin D supplementation on serum total 25(OH)D and directly measured free 25(OH)D

**DOI:** 10.1530/EJE-15-1089

**Published:** 2016-04

**Authors:** Stina T Sollid, Moira Y S Hutchinson, Vivian Berg, Ole M Fuskevåg, Yngve Figenschau, Per M Thorsby, Rolf Jorde

**Affiliations:** 1 Tromsø Endocrine Research Group, Department of Clinical Medicine, UiT The Arctic University of Norway, Tromsø, Norway; 2 Division of Internal Medicine, University Hospital of North Norway, 9038, Tromsø, Norway; 3 Division of Head and Motion, Department of Rheumatology, Nordland Hospital, Bodø, Norway; 4 Division of Diagnostic Services, University Hospital of North Norway, Tromsø, Norway; 5 Department of Medical Biology, UiT The Arctic University of Norway, Tromsø, Norway; 6 Hormone Laboratory, Department of Medical Biochemistry, Oslo University Hospital, Oslo, Norway

## Abstract

**Objective:**

To determine the relationship between serum total 25-hydroxyvitamin D (25(OH)D), directly measured free 25(OH)D and calculated free 25(OH)D with regard to vitamin D-binding protein (DBP) phenotypes, sex, BMI, age and season, and their interrelationship to vitamin D supplementation.

**Design, patients and interventions:**

A randomized controlled trial with 20 000 IU of vitamin D_3_ per week or placebo for 12 months was designed. A total of 472 subjects, 236 in each of the intervention groups, were included in the analyses.

**Main outcome measures:**

Baseline serum concentrations and increases in serum total 25(OH)D, directly measured free 25(OH)D, calculated free 25(OH)D and DBP.

**Results:**

Serum total 25(OH)D and DBP concentrations were significantly lower in subjects with the phenotype Gc2/Gc2 compared to phenotypes with the Gc1S allele, and lower in males compared to females. When using directly measured free 25(OH)D, the differences related to DBP phenotypes and sexes were clearly diminished. All calculated free 25(OH)D concentrations were overestimated compared to the directly measured free 25(OH)D. Serum parathyroid hormone showed an inverse correlation with all vitamin D parameters analyzed. The increases after 12 months of vitamin D supplementation were not significantly different for any of the vitamin D parameters regardless of DBP phenotype, sex or age. Supplementation with vitamin D did not affect serum DBP.

**Conclusion:**

Direct measurements of free 25(OH)D reduce the differences seen in total 25(OH)D between DBP phenotype groups and sexes, probably caused by differences in DBP concentrations. With conditions affecting serum DBP concentrations, direct measurements of free 25(OH)D should be considered.

## Introduction

Total 25-hydroxyvitamin D (25(OH)D) is the metabolite used to evaluate a person's vitamin D status. In the circulation close to 90% of total 25(OH)D are bound to vitamin D-binding protein (DBP) with high affinity, about 10% are more loosely bound to albumin and <0.1% are in an unbound, or free, form (1). For the calculation of free 25(OH)D one therefore needs to know serum total 25(OH)D concentrations as well as serum DBP and albumin concentrations (2). Recently it has also been possible to measure free 25(OH)D directly using a commercially available kit. According to the ‘free hormone hypothesis’ it is the unbound hormone that is the biologically active; for 25(OH)D this may also include the fraction bound to albumin that together with the unbound form have been classified as ‘bio-available’ 25(OH)D (3).

DBP is synthesized by the liver, a process that is stimulated by estrogen (4). Some conditions, such as cirrhosis due to affected synthesis (5) and nephritic syndrome due to protein loss (4) are associated with low DBP concentrations, while pregnancy (6) and estrogen therapy (7) are both known to cause higher DBP concentrations. More than 120 genetic variations of DBP exist; however, for practical purposes the three major polymorphic alleles of DBP in humans, GC1F, GC1S and GC2, yielding six allelic combinations and corresponding phenotypes, are the relevant ones (8). These genetic factors have been showed to explain almost 80% of the variations in serum DBP concentrations, which show great differences between ethnic groups (9). Thus, in Europeans the Gc1S allele is most frequently seen, whereas in Africans GC1F is the most common allele (4).

In spite of lower serum total 25(OH)D concentrations African Americans have better bone health and less fractures than European Americans (10). However, Powe *et al*. (9) have shown that the known differences in serum total 25(OH)D between African and European Americans equalizes when evaluating calculated free or bio-available 25(OH)D concentrations, and differences in serum total 25(OH)D may therefore not reflect differences in biological activity. Recently this finding in African and European Americans was confirmed by Aloia *et al*. (11) using directly measured free serum 25(OH)D. Accordingly, other differences in serum total 25(OH)D, like those between DBP phenotypes, sexes, age groups, BMI categories, as well as seasons, may diminish or disappear when evaluating free 25(OH)D concentrations. On the other hand, relations with presumed vitamin D related effects, like parathyroid hormone (PTH) secretion, might increase.

Furthermore, there are conflicting reports on the DBP response to vitamin D supplementation (12, 13). If a response exists, that would affect not only the total 25(OH)D, but to an even greater degree the free 25(OH)D concentrations. We have recently performed a large randomized controlled trial (RCT) with high dose vitamin D supplementation where we have genotyped for DBP polymorphisms, have calculated and directly measured concentrations of free serum 25(OH)D, and therefore had the opportunity to address the above questions.

## Subjects and methods

### Overall design

The data in the present study (baseline and 12 months) were obtained from an RCT where vitamin D vs placebo was given to subjects with prediabetes for 5 years with prevention of diabetes as primary endpoint. Included in the analysis are those with complete datasets for serum albumin, serum total 25(OH)D, serum directly measured free 25(OH)D and serum DBP.

### Study population

Methods regarding the conduct of the RCT have been published in detail previously (14). Briefly, inclusion criteria were 21–80 years of age and impaired fasting glucose (IFG) and/or impaired glucose tolerance (IGT). Exclusion criteria were primary hyperparathyroidism, sarcoidosis or other granulomatous disorders, urolithiasis, cancer during the last 5 years, reduced kidney function, or unstable angina pectoris, acute myocardial infarction or stroke the last year. Fertile women had to use contraception, could not be pregnant, and could not be lactating. Study participants were allowed to take vitamin D supplements of maximum 400 IU/day. At baseline 511 subjects received study medication, 256 subjects were given 20 000 IU of vitamin D_3_ per week and 255 placebo. Four hundred eighty-four, 242 in each group, completed the 1-year visit.

### Measurements

Height and weight were measured wearing light clothing and no shoes. BMI was calculated as weight (kg) divided by squared height (m^2^). Serum calcium, serum PTH and HbA_1c_ were measured as previously described (15). Serum total 25(OH)D was measured by an in-house LC–MS/MS, the limit of detection (LoD) was <4 mmol/l, and the between day coefficient of variation (CV) <9% (16). Serum albumin was measured by a colorimetric method (bromocresol green) using an automated analyzer, Cobas 8000 (c702, Roche Diagnostics). Serum DBP was measured by using an in house competitive RIA with a polyclonal antibody according to Kauppinen-Mäkelin *et al*. (17) at the Hormone Laboratory, Oslo University Hospital. Direct measurement of serum free 25(OH)D was done using a competitive ELISA kits from Diasource Diagnostics based on patented MAB developed by Future Diagnostics (http://www.vitamin-d-diagnostics.com/Vitamin-D/Free-25OH-Vitamin-D/Free-25OH-Vitamin-D-ELISA-96-tests-RUO), the range was 0.2–87.4 pmol/l, the LoD was 7.0 pmol/l and the precision was <10%.

### Genotyping and calculations of free and bio-available 25(OH)D

Genotyping was performed by KBiosciences (http://www.lgcgenomics.com/genotyping/) with a competitive allele-specific PCR (KASPar) assay. Based on genotyping for two single-nucleotide polymorphisms (SNPs) in the DBP gene (rs7041 and rs4588), the six phenotypes were identified (Supplementary Tables 1 and 2, see section on supplementary data given at the end of this article). For serum 25(OH)D the binding coefficient for albumin is 6×10^5^/M and the binding coefficient for DBP is 7×10^8^/M (2). Free- and bio-available serum 25(OH)D concentrations were calculated using serum total 25(OH)D, DBP and albumin concentrations using a free testosterone equation (18) adapted for calculating free 25(OH)D (19) (Supplementary material).

### Statistical analysis

Normal distribution was evaluated by visual inspection of histograms and by skewness and kurtosis. Normally distributed data are presented as mean±s.d., PTH being the only non-normally distributed variable is presented as median (2.5th and 97.5th percentiles). Level of significance was set at *P*<0.05 (two-tailed). Independent samples *t*-test was used to compare the vitamin-D and placebo groups, whereas paired samples *t*-tests were used to compare serum calculated free 25(OH)D and directly measured free 25(OH)D. For sex *χ*^2^ test and for age and BMI one-way ANOVA were used to determine differences in distribution between DBP phenotype groups, BMI groups and age groups. A general linear model was used to examine the DBP phenotype – vitamin D parameters associations with sex, BMI, age and season as covariates. The Bonferroni procedure was used for *post-hoc* analysis. Independent sample *t*-tests were used to evaluate differences between sex and season, whereas linear trend analyses were used across BMI groups and age groups. Univariate correlations were assessed with Pearson correlation coefficient (PTH was log transformed). IBM SPSS Statistics version 22 was used for all statistical analyses.

### Ethics

The study was approved by the Norwegian Medicines Agency and by the Regional Committee for Medical Research Ethics. The trial is registered at ClinicalTrials.gov (NCT00685594).

## Results

### Participant characteristics

Complete data sets were available in 472 subjects, 236 in each of the intervention groups. Baseline values are presented in Table 1. The directly measured free 25(OH)D concentrations were lower than the calculated free 25(OH)D (Table 1). There were no significant differences between the vitamin D and placebo groups.

### Serum total 25(OH)D, calculated free 25(OH)D, calculated bio-available 25(OH)D, directly measured free 25(OH)D and DBP in relation to DBP phenotypes at baseline

As expected, Gc1S was by far the most abundant allele. There were no significant differences between the DBP phenotypes regarding sex, BMI, age or albumin (Table 2).

Serum total 25(OH)D concentrations were significantly lower for the phenotype Gc2/Gc2 compared to Gc1S/Gc1S, Gc1S/Gc1F and GC1S/Gc2 (Table 2). The phenotype Gc2/Gc2 also had significantly lower serum DBP concentration compared with all the other phenotype groups (Table 2).

For calculated free 25(OH)D, calculated bio-available 25(OH)D and directly measured free 25(OH)D the differences between the DBP phenotypes diminished and were no longer statistically significant (Table 2). The least differences between phenotypes were found for the direct measurements; as an example the difference between Gc2/Gc2 and Gc1S/Gc1S were 30.2% for total 25(OH)D, 17.6% for calculated free 25(OH)D and only 9.0% for directly measured free 25(OH)D.

We also calculated free 25(OH)D using specific binding coefficients for the six different DBP phenotypes; however, this did not improve the results (data not shown).

### Serum total 25(OH)D, calculated free 25(OH)D, calculated bio-available 25(OH)D, directly measured free 25(OH)D and DBP in relation to sex, BMI, age and season at baseline

Males had significantly lower serum total 25(OH)D than females, a difference of 10.5% (Supplementary Table 3, see section on supplementary data given at the end of this article). Similarly, serum DBP concentrations were significantly lower for males compared to females; and in line with this no significant differences were found for serum calculated free 25(OH)D, calculated bio-available 25(OH)D or directly measured free 25(OH)D concentrations (Supplementary Table 3).

With increasing BMI a non-significant trend (*P*=0.08) for decreasing serum total 25(OH)D was seen with a 9.2% difference between the lowest and highest BMI groups. A similar, but significant trend was seen for serum DBP. As for total 25(OH)D there were no significant differences in the free and bio-available 25(OH)D concentrations between BMI groups; further, for the directly measured free 25(OH)D the difference had almost completely disappeared (Supplementary Table 3).

There was with increasing age a significant linear trend with increasingly higher serum total 25(OH)D, calculated free 25(OH)D, calculated bio-available 25(OH)D and directly measured free 25(OH)D (Supplementary Table 3). However, no such trend was seen for serum DBP, and therefore the differences between the lowest and highest age groups for total 25(OH)D and the corresponding differences for directly measured free 25(OH)D were basically the same (17.0% and 17.1% respectively).

For season we compared the three consecutive highest months of serum total 25(OH)D (July–September) with the three lowest (February–April), and since their serum DBP did not differ significantly, the difference remained statistically significant, and also almost identical, for the calculated free 25(OH)D, calculated bio-available 25(OH)D and directly measured free 25(OH)D (*P*<0.001) (data not shown).

### Correlations for serum total 25(OH)D, calculated free 25(OH)D, directly measured free 25(OH)D, albumin, DBP and PTH at baseline

Serum PTH was negatively and similarly correlated with serum total 25(OH)D and directly measured free 25(OH)D (Table 3). Additionally, strong correlations were found between serum total 25(OH)D and directly measured free 25(OH)D (Table 3), and also between all other vitamin D parameters (data not shown). Serum DBP correlated with total 25(OH)D, but not the directly measured free 25(OH)D (Table 3).

### Effect of vitamin D supplementation on serum DBP, total 25(OH)D, calculated free 25(OH)D and directly measured free 25(OH)D

In both the vitamin D and the placebo group there was a slight and similar non-significant decrease in serum DBP after 1 year (−0.2±0.5 and −0.2±0.6 μmol/l respectively). Similarly, there were no statistically significant differences between the six DBP phenotypes, sexes, BMI groups or age groups regarding change in serum DBP (Supplementary Tables 4 and 5, see section on supplementary data given at the end of this article). Further, there were no significant differences between DBP phenotypes, sexes or age groups in changes in serum total 25(OH)D, calculated free 25(OH)D or directly measured free 25(OH)D (Supplementary Tables 4 and 5). Even so, there appeared to be non-significant differences in the increase in serum total 25(OH)D between DBP groups phenotype (Supplementary Table 4). For all 25(OH)D measures the highest increases were seen for subjects in the lowest BMI groups (Supplementary Table 5).

**Figure 1:**
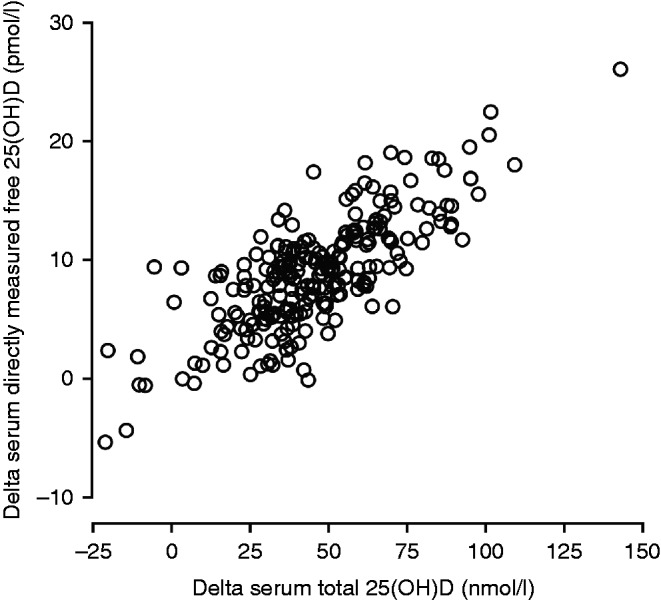
Correlation between delta (12 month values minus baseline value) serum total 25(OH)D and delta directly measured free 25(OH)D.

There was a strong correlation between increase in all measures of serum 25(OH)D as exemplified between delta serum total 25(OH)D and delta serum directly measured free 25(OH)D in Fig. 1. The correlation was similar for all six DBP phenotypes (data not shown).

## Discussion

In the present study we have found that serum total 25(OH)D concentrations are dependent on DBP phenotype, that differences between DBP phenotypes diminish when calculating or measuring serum free 25(OH)D, and that serum DBP concentrations are unaffected by vitamin D supplementation. Further, calculations appear to overestimate the free 25(OH)D concentrations compared to the direct measurements.

Serum total 25(OH)D concentrations were significantly lower for the DBP phenotype Gc2/Gc2 compared to phenotypes with the Gc1S allele as previously reported (20). Although the serum calculated free 25(OH)D, calculated bio-available 25(OH)D and directly measured free 25(OH)D also were lowest in this phenotype, the differences were greatly diminished and no longer significant. Thus, the difference between the DBP phenotypes Gc2/Gc2 and Gc1S/Gc1S decreased from 30.2% for total 25(OH)D to 9.0% for directly measured free 25(OH)D. This is in line with the almost identical free 25(OH)D concentrations in black and white Americans (9, 11), despite great differences in total 25(OH)D, due to different distribution of DBP phenotypes (4). This could also partly explain why African Americans have better bone health than European Americans despite lower total 25(OH)D concentrations.

It is well known that serum DBP concentrations are affected by phenotype (21), and in our study serum DBP concentrations for the phenotype Gc2/Gc2 were significantly lower compared to all other phenotypes. This has also been found in most (20, 22, 23), but not all previous studies (9). Thus, Powe *et al*. (9) found that the DBP phenotype Gc1F/Gc1F, predominantly found in black Americans, had by far the lowest serum DBP concentration. This is contrary to our study, and other studies from Scandinavia (21, 22), where this phenotype had the highest serum DBP concentration. As reported by Bouillon *et al*. the discrepancy may be due to differences in antibodies used in the DBP assays (24), or perhaps it reflects that there are other race-related factors than DBP phenotype that affect the serum DBP concentration.

Males had significantly lower serum total 25(OH)D concentrations and also serum DBP concentrations than females. For calculated free 25(OH)D and calculated bio-available 25(OH)D these differences between the sexes diminished, and for the directly measured free 25(OH)D the concentrations were equal. The difference in serum DBP between sexes has previously been reported (25) and is most likely caused by the estrogen susceptible DBP synthesis (4). Similar, although non-significant, the difference between the lowest and highest BMI group for serum total 25(OH)D concentrations were reduced for the directly measured free 25(OH)D, probably because of the significant linear trend with lower serum DBP concentration with higher BMI. For BMI earlier reports are conflicting with some reporting differences in serum DBP (26), while others report no differences (25, 27). This question is therefore not settled.

As in previous reports from our region (28, 29), there was for age groups a significant linear trend with the highest serum total 25(OH)D concentrations in the oldest subjects. This is most likely due to a diet richer in fatty fish and a higher use of vitamin D supplements in the oldest age groups (28). However, serum DBP did not differ between age groups, and a significant positive linear trend was also seen for all the other 25(OH)D measures.

Similar to that seen for age group, there were no differences in serum DBP between the months with the highest and lowest serum total 25(OH)D concentrations, and accordingly, the differences between these months remained for serum calculated free 25(OH)D, calculated bio-available 25(OH)D and directly measured free 25(OH)D.

We found that the calculated serum free 25(OH)D concentrations are overestimated compared to the directly measured free 25(OH)D. For calculations of serum free 25(OH)D concentration DBP concentrations are needed. Since different DBP assays recognize the DBP phenotypes differently (11, 24), this will affect the calculated free 25(OH)D concentrations. Furthermore, the validity of the equation for calculating free 25(OH)D concentration which is derived from an equation for free testosterone (2), has been questioned (24). Therefore, it may be wise not to use the calculated concentrations before these issues have been settled.

We also performed analyses with the DBP phenotype specific binding coefficients. However, and contrary to what we expected, this did not improve the results. The binding coefficients for Gc1S, Gc1F and GC2 are the binding coefficients for the homozygote DBP phenotypes; however, the binding coefficients used for the heterozygote DBP phenotypes were the mean of the two combined haplotypes binding coefficient (30). These binding coefficients may not be correct, which may at least partly explain our unexpected findings.

Since vitamin D both indirectly, through increased calcium absorption from the intestines, and directly, through inhibition of PTH synthesis, decreases the PTH secretion, the serum PTH concentrations has been suggested as a vitamin D biomarker (31). One could therefore hypothesize that the 25(OH)D measure that best correlate with the serum PTH concentrations would be the best indicator of the vitamin D status. Unfortunately, previous studies are conflicting; Schwartz *et al*. (32) reported a significant inverse correlation with PTH for directly measured free 25(OH)D, but not for calculated free 25(OH)D, whereas Aloia *et al*. (11) in one study reported a significant negative correlation with PTH for total 25(OH)D, but not for directly measured free 25(OH)D. However, in another study by Aloia *et al*. (33) a significantly negative correlation for both total 25(OH)D and directly measured free 25(OH)D was found, and in our study, all measures of 25(OH)D had a similar and negative correlation with PTH. Although the free hormone hypothesis is appealing, it should be recalled that there is a receptor-mediated endocytosis of the DBP/total 25(OH)D complex in the proximal renal cells (34), and probably also in other cells (1), which could favor serum total 25(OH)D as an indicator of vitamin D status.

As compared to placebo, there was no effect on the serum DBP concentrations by vitamin D supplementation for 1 year regardless of DBP phenotype, sex, BMI or age group which is similar to that reported by Ponda *et al*. (13) and Sonderman *et al*. (35). Similarly, in the vitamin D group there were no significant differences in increase for any of the vitamin D parameters between the DBP phenotypes, sexes or age groups. On the other hand, there appear to be non-significant differences between the DBP phenotype groups regarding the increase in serum total 25(OH)D; however, the DBP phenotype groups are small and thereby limits the power to detect any real differences. It therefore appears unlikely that the DBP concentration is regulated in order to keep the free fraction of serum 25(OH)D stable as seen in other endocrine systems (36).

There are some limitations to our study; we included subjects with prediabetes which limits the generalizability of the results, the study population consists almost exclusively of Caucasians leaving us with a skewed distribution of DBP phenotypes, and we did not have bone density measurements that could have given additional information regarding which 25(OH)D parameter is the best biomarker of vitamin D status. On the other hand, we included a large number of subjects, had direct measurements of serum free 25(OH)D, and could also examine the effects of vitamin D supplementation on different vitamin D parameters and serum DBP, which so far have not been thoroughly studied.

In conclusion, we have found that the direct measurement of free 25(OH)D diminishes differences between DBP phenotypes and sexes as compared to serum total 25(OH)D. In situations where DBP phenotypes differ between groups, as seen between white and black Americans, and in conditions associated with low DBP concentrations like liver cirrhosis or nephritic syndrome, or high DBP concentrations like pregnancy and estrogen therapy, direct measurement of serum free 25(OH)D should be considered.

## Supplementary data

This is linked to the online version of the paper at http://dx.doi.org/10.1530/EJE-15-1089.

## Author contribution statement

R Jorde is the guarantor of this work and had full access to all the data in the study and takes responsibility for the integrity of the data and the accuracy of the data analysis. S T Sollid researched data and wrote the manuscript; M Y S Hutchinson researched data; all authors contributed to the discussion, review and editing of the manuscript.

## Figures and Tables

**Table 1:** Baseline characteristics of all subjects and according to randomization status.

	**All subjects**	**Randomization status**	
Vitamin D	Placebo
Number of subjects	472	236	236
Male/female	293/179	150/86	143/93
BMI (kg/m^2^)	29.9±4.3	30.1±4.2	29.8±4.4
Age (years)	62.0±8.7	62.0±8.2	62.0±9.1
HbA_1c_ (%)	6.0±0.3	6.0±0.3	6.0±0.3
Serum calcium (mmol/l)	2.31±0.08	2.31±0.07	2.31±0.08
Serum PTH (pmol/l)	5.3 (5.5, 5.9)	5.5 (5.5, 6.1)	5.3 (5.3, 5.9)
Serum albumin (g/l)	45.2±2.3	45.2±2.3	45.1±2.2
Serum DBP (μmol/l)	3.7±0.5	3.7±0.5	3.7±0.6
Serum total 25(OH)D (nmol/l)	60.7±21.6	59.9±22.0	61.5±21.1
Serum calculated free 25(OH)D (pmol/l)	20.4±7.0	20.4±7.2	20.4±6.7
Serum calculated bio-available 25(OH)D (nmol/l)	8.3±2.8	8.3±3.0	8.3±2.6
Serum directly measured free 25(OH)D (pmol/l)	13.7±4.2	13.7±4.3	13.7±4.2

PTH, parathyroid hormone; DBP, vitamin D binding protein; 25(OH)D, 25-hydroxyvitamin D.

**Table 2:** Distribution of sex, age, BMI, albumin, DBP and vitamin D parameters according to DBP phenotype. General linear model with sex, BMI, age and season as covariates. Bonferroni method for *post-hoc* analysis.

	**DBP phenotypes**					
Gc1S/Gc1S	Gc1S/Gc1F	Gc1S/Gc2	Gc1F/Gc1F	Gc1F/Gc2	Gc2/Gc2
Number of subjects	148	124	117	20	39	24
Sex (females/males)	58/90	53/71	37/80	7/13	16/23	8/16
BMI (kg/m^2^)	29.3±4.5	30.2±4.2	30.4±4.2	31.3±4.0	29.7±4.6	30.1±2.9
Age (years)	62.2±8.4	61.3±9.2	62.4±8.4	63.3±8.9	61.3±9.0	62.4±9.3
PTH (pmol/l)	5.6 (5.2, 5.9)	6.0 (5.5, 6.4)	5.9 (5.4, 6.3)	5.3 (4.6, 5.9)	5.4 (5.0, 5.7)	5.2 (4.5, 5.8)
Serum albumin (g/l)	45.1±2.3	45.3±2.0	45.1±2.5	45.5±2.0	45.4±2.2	45.4±2.5
Serum DBP (μmol/l)	3.7±0.5^b^	3.8±0.5^b^	3.6±0.5^b^	3.9±0.7^b^	3.8±0.7 ^b^	3.1±0.4 ^a^
Serum total 25(OH)D (nmol/l)	62.9±23.9^b^	64.2±22.9^b^	59.6±19.6^d^	56.8±17.0	56.9±17.7	43.9±13.5 ^a^,^c^
Serum calculated free 25(OH)D (pmol/l)	21.0±7.4	21.1±6.9	20.4±6.8	18.6±6.4	18.8±6.2	17.3±5.5
Serum calculated bio-available 25(OH)D (nmol/l)	8.6±3.1	8.7±2.8	8.3±2.7	7.7±2.7	7.7±2.6	7.1±2.2
Serum directly measured free 25(OH)D (pmol/l)	13.4±4.2	14.2±4.3	13.9±4.2	12.4±3.3	14.0±4.6	12.2±4.1

DBP, vitamin D binding protein; 25(OH)D, 25-hydroxyvitamin D.

a Significantly lower than *P*<0.001.

b *P*<0.001.

c Significantly lower than *P*<0.05.

d *P*<0.05.

**Table 3:** Correlations between variables.

	**Serum total 25(OH)D** (nmol/l)	**Serum directly measured free 25(OH)D** (pmol/l)	**Serum DBP** (μmol/l)	**Serum albumin** (g/l)	**Serum PTH** (pmol/l)
Serum total 25(OH)D (nmol/l)	1	0.73*	0.24*	−0.04	−0.15*
Serum directly measured free 25(OH)D (pmol/l)	0.73*	1	0.05	−0.05	−0.12*
Serum DBP (μmol/l)	0.24*	0.05	1	0.15*	−0.04
Serum albumin (g/l)	−0.04	−0.05	0.15*	1	−0.06^†^
Serum PTH (pmol/l)^a^	−0.21*	−0.17*	−0.05	−0.09	1

*P<0.001; ^†^*P*<0.05; 25(OH)D, 25-hydroxyvitamin D; DBP, vitamin D binding protein; PTH, parathyroid hormone.

a Denotes log-transformed data.
